# Monophyly, Distance and Character–Based Multigene Barcoding Reveal Extraordinary Cryptic Diversity in *Nassarius*: A Complex and Dangerous Community

**DOI:** 10.1371/journal.pone.0047276

**Published:** 2012-10-11

**Authors:** Shanmei Zou, Qi Li, Lingfeng Kong

**Affiliations:** 1 Key Laboratory of Mariculture Ministry of Education, Ocean University of China, Qingdao, China; 2 Key Laboratory of Mariculture Ministry of Education, Ocean University of China, Qingdao, China; 3 Key Laboratory of Mariculture Ministry of Education, Ocean University of China, Qingdao, China; Auburn University, United States of America

## Abstract

**Background:**

Correct identification and cryptic biodiversity revelation for marine organisms are pressing since the marine life is important in maintaining the balance of ecological system and is facing the problem of biodiversity crisis or food safety. DNA barcoding has been proved successful to provide resolution beyond the boundaries of morphological information. *Nassarius*, the common mudsnail, plays an important role in marine environment and has problem in food safety, but the classification of it is quite confused because of the complex morphological diversity.

**Methodology/Principal Findings:**

Here we report a comprehensive barcoding analysis of 22 *Nassarius* species. We integrated the mitochondrial and nuclear sequences and the morphological characters to determine 13 *Nassarius* species studied and reveal four cryptic species and one pair synonyms. Distance, monophyly, and character–based barcoding methods were employed.

**Conclusions/Significance:**

Such successful identification and unexpected cryptic discovery is significant for *Nassarius* in food safety and species conversation and remind us to pay more attention to the hidden cryptic biodiversity ignored in marine life. Distance, monophyly, and character–based barcoding methods are all very helpful in identification but the character-based method shows some advantages.

## Introduction

It is pressing to catalogue the earth’s species since the world is facing a global biodiversity crisis [1], [2]. The rapid loss of marine biodiversity has prompted efforts to catalogue the biodiversity, such as the Census of Marine Life (www.coml.org). Large numbers of marine organisms are important in maintaining the balance of ecological system, and many of them are consumed as seafood. Thus, correct species identification and revelation of cryptic species diversity for marine life is important to nature conversation, food safety and better understanding the patterns of ecosystem functioning. Nevertheless, due to the declining number of taxonomists [3], the insufficient funding for taxonomy and the confused morphological diversity, it is hard for traditional taxonomy to undertake the huge taxonomic task for marine organisms.

While the traditional taxonomy has been declining, DNA-based techniques, such as DNA barcoding [4], often provide resolution beyond the boundaries of morphological information [5]. DNA barcoding, which involves taxon identification using standardized DNA regions, has recently received much attention [6], [7], [8], [9]. It is an aid to the discrimination and identification of species and can recover new or cryptic species [10], [11]. Until now, DNA barcoding has been successfully applied to many animals (e.g. [12], [13], [14], [15], [16], [17]). Two broad methods of DNA barcoding (distance and monophyly-based methods) have been originally used. Distance-based method is based on the “barcoding gap”, the degree of DNA sequence variation within and between species. Monophyly-based method requires the recovery of species as discrete clades (monophyly) on a phylogenetic tree [6]. Nevertheless, some issues complicate the use of both methods [18], [19], [20], [21], [22], [23]. A recently applied new technique, the character-based DNA barcode approach, characterizes species through a unique combination of diagnostic characters [18], [23], [24], [25] and has been proved useful for species identification and discovery of cryptic species [15], [23], [24].

Nassariidae is a large gastropod group, comprising about 300 extant and almost 600 extinct nassariid species that are organized into 12 genera and 31 subgenera [26]. Three nassariid subfamilies are commonly recognized [26], [27]: the Dorsaninae, the Cylleninae and the cosmopolitan Nassariinae. *Nassarius*, the common mudsnail, is a species-rich genus of Nassariinae and is distributed throughout worldwide oceans [28]. The nassariids of *Nassarius* are usually less than 50 mm in adult shell height [29]. Ecologically, most nassariids of *Nassarius* are thought to be facultative scavengers inhabiting inter- to subtidal shallow marine environments [26]. As scavengers, nassariids of *Nassarius* are important in maintaining the balance of ecological system, especially for the balance of benthic community. They are also useful in the biomonitoring of Tributyltin (TBT) pollution in marine environment. Due to the high specificity and sensitivity to TBT, imposex phenomenon is found in some *Nassarius* species. In fact, imposex is considered the best biological indicator of TBT pollution in marine waters [30]. More importantly, food safety problem exists in *Nassarius*. Most species of *Nassarius* are consumed as food in China where they are widely distributed. Nevertheless, maybe due to the food nassariids of *Nassarius* get from marine waters, different toxins are concentrated in *Nassarius* sp’s body. Recent studies find that the toxicity of *Nassarius* is relative to species [31], [32]. For example, *N. hepaticus* are toxic gastropods, *N. festiva* are non-toxic gastropods, while the toxicity of *N. succinctus* probably change with the season [32].

Despite the importance of maintaining the balance of ecological system, the usefulness of monitoring TBT pollution and the danger of eating, the taxonomy of *Nassarius* species is still confusing. Discrimination of the *Nassarius* species is mainly based on the shell morphology, especially the sculpture [33]. However, due to the intraspecific shell variation affected by biotic and abiotic factors [34], [35] and the various shell forms in different species, the identification of *Nassarius* species is often difficult. Environment adaptive intraspecific morphological variation can lead to ambiguous identification of closely related species [36], and interspecific uniformity may also present difficulties in species identification [37]. Thus, it is arbitrary to identify *Nassarius* species only using morphological characters and there is probably some misidentification and a significant amount of cryptic diversity within *Nassarius*. Some *Nassarius* species that are considered as single may be erroneously classified under one species name. Unfortunately, until now there are few large-scale reliable genetic studies to identify *Nassarius* species and estimate the level of cryptic diversity within *Nassarius*. Li *et al.* [38] employed mitochondrial sequences to study the identification and phylogeny of *Nassarius*. Nevertheless, due to the very limited samples, the status of *Nassarius* species is still unclear.

In this study we reported a barcoding analysis of 22 *Nassarius* species. Many of the species have diverse morphological characters and are easily confused. Two mitochondrial genes COI and 16S rRNA and one nuclear gene ITS-1 were employed. Distance, monophyly, and character–based barcoding methods were conducted. We integrated the molecular and morphological data: (1) to identify the species and reveal the cryptic diversity within *Nassarius* (2) to test the performance of DNA barcoding and three barcode methods for morphologically complex species.

## Materials and Methods

### Ethics Statement

No specific permits were required for the described field studies. The field studies did not involve endangered or protected species. No specific permissions were required for the locations. The locations are not privately-owned or protected in any way.

### Sample Collections

**Figure 1:**
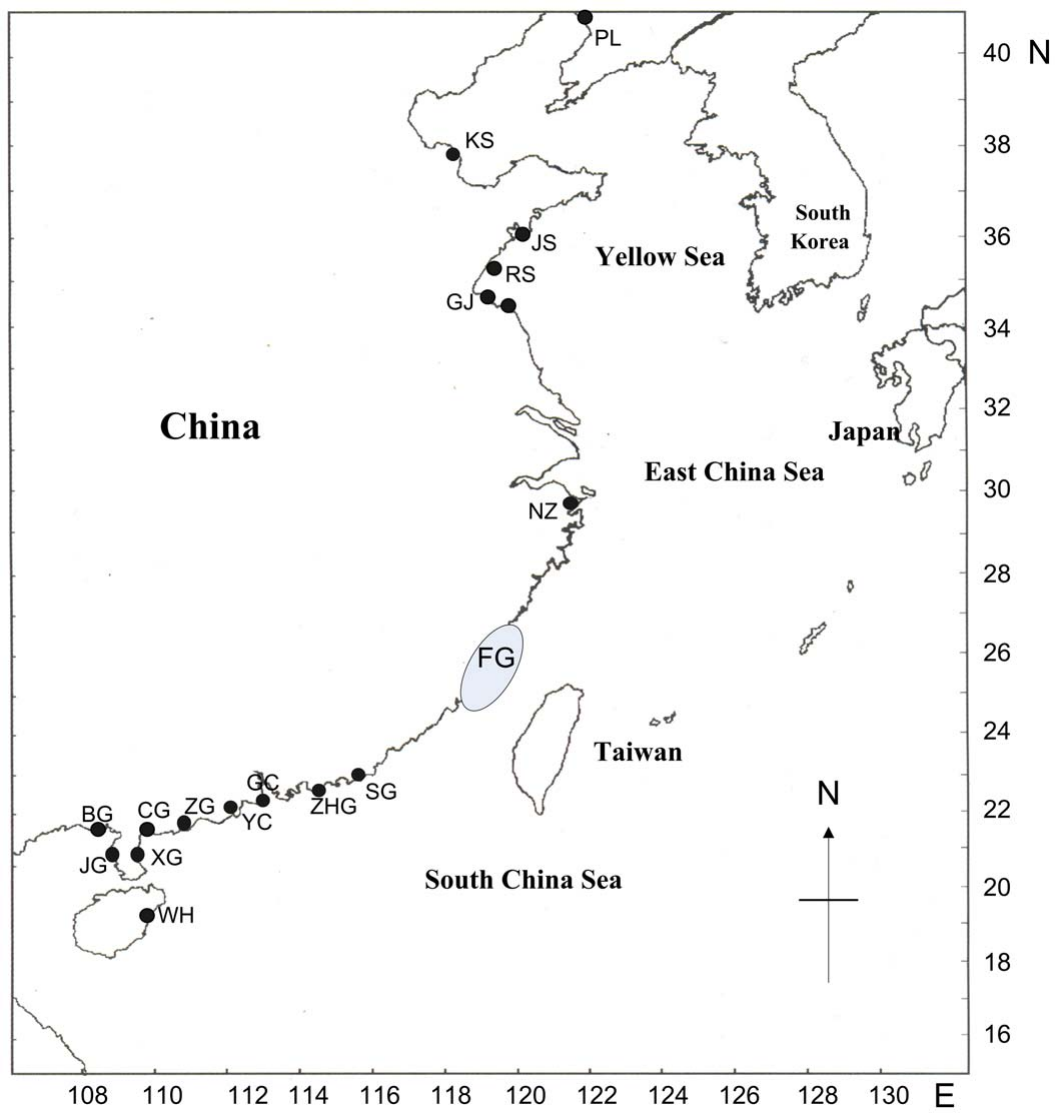
Sampling sites in this study. The letter codes correspond to geographic locations listed in table S1.

A total of 220 samples representing 22 *Nassarius* species were used in this study (Table S1). Thereinto, 208 specimens were collected across the whole China coast from 2005 to 2011 (Figure 1). One or more specimens were chosen from each locality in order to include as many morphologically distinguishable individuals per site as possible. Specimens were collected and stored in 90–100% ethanol.

### DNA Extraction, PCR Amplification and Sequencing

DNA was extracted from small pieces of foot tissue by the CTAB method as modified by Winnepenninckx *et al.* [39]. PCR reactions were carried out in a total volume of 50 µL, using 1.5 mM MgCl_2_, 0.2 mM of each dNTPs, 1 µM of both forward and reverse PCR primers, 10× buffer and 2.5 U *Taq* DNA polymerase. Thermal cyclings were performed with an initial denaturation for 3 min at 95°C, 45 s at primer-specific annealing temperatures, and 1 min at 72°C, followed by 35 cycles of 30 s at 95°C, 45 s at primer-specific annealing temperatures, 1 min at 72°C, with a final extension of 10 min at 72°C. PCR and sequencing primers for COI, 16S rRNA and ITS-1 genes were listed in Table 1. The PCR products were confirmed by 1.5% agarose gel electrophoresis and stained with ethidium bromide. The fragment of interest was purified using EZ Spin Column PCR Product Purification Kit, Sangon. Purified products were sequenced in both directions using the BigDye Terminator Cycle Sequencing Kit (ver. 3.1, Applied Biosystems) and an AB PRISM 3730 (Applied Biosystems) automatic sequencer.

**Table 1:** Sequences of the primers used in the PCRs.

Name	Sequence 5′-3′	Annealing Temperature (^o^C)	Source
COI			
LCO1490 (F)	GGTCAACAAATCATAAAGATATTGG	45–50	[56]
HCO2198 (R)	TTAACTTCAGGGTGACCAAAAAATCA	45–50	[56]
16S			
16Sar	CGCCTGTTTATCAAAAACAT	51	[57]
16Sbr	CCGGTCTGAACTCAGATCACGT	51	[57]
16SarM	GCGGTACTCTGACCGTGCAA	48–50	[16]
16SbrM	TCACGTAGAATTTTAATGGTCG	48–50	[16]
ITS-1			
ITS-1 (F)	TAACAAGGTTTCCGTAGGTGAA	52	[58]
ITS-1 (R)	GCTGCGTTCTTCATCGATGC	52	[59]

### Distance and Phylogenetic Analysis

Forward and reverse sequences of each gene were edited, assembled and merged into consensus sequences using the software program Sequencher 4.5 (Genecodes Corporation, Ann Arbor, MI). Sequences were aligned using the program, fftnsi, which is implemented in MAFFT 6.717 [40]. Alignment of COI nucleotide sequences was unproblematic since indels were absent. For 16S rDNA and ITS-1 sequences, areas of uncertain alignment were omitted by the software Gblocks 0.91b [41], with minimum number of sequences for a conserved position set to 50% of the total, minimum number of sequences for a flanking position set to 90% of the total, maximum number of contiguous non-conserved positions set to 3, minimum length of a block set to 5, and half gap positions allowed.

For distance analyses, pairwise sequence divergences were calculated using a Kimura 2-parameter (K2P) distance model in MEGA 4.0 [42]. Phylogenetic analysis of COI, 16S rDNA and ITS-1 sequences were carried out using neighbour joining (NJ) and Bayesian methods. The species *Fusinus longicaudus*, *Euplica scripta*, *Mitrella burchardi* and *Pseudamycla formosa* were selected as the outgroups. NJ analyses were conducted using K2P distance model as recommended by Hebert et al. [4] in MEGA 4.0 [42]. Bayesian analyses were carried out using the Monte Carlo Markov Chainmethod (MCMC) implemented on MrBayes v.3.1.2 [43]. Nucleotide substitution models for Bayesian analyses were selected separately for each gene using the Akaike Information Criterion (AIC) as implemented in the jModeltest v.0.1.1 [44]. The most appropriate models for Bayesian analyses were HKY+I+G for COI, HKY+I+G for 16S and GTR+G for ITS-1. Four chains were run twice in parallel for 10^7^ generations, and trees were sampled very 100 generations. Stationarity was considered to be reached when the average standard deviation of split frequencies shown in MrBayes were less than 0.01 [43]. Chain convergence was further verified by ensuring potential scale reduction factors neared 1 and using Tracer v.1.5 to confirm sufficiently large ESS values. Burn-ins were determined by visually inspecting the –ln *L* trace plot in Tracer.

### Character-Based Barcode Analysis

The characteristic attribute organization system (CAOS) [45], [46] was used for the character-based identification method. The CAOS algorithm identifies character-based diagnostics, here termed “characteristic attributes” (CAs), for every clade at branching node within a guide tree that is first produced from a given dataset. The system comprises two programs: P-Gnome and P-Elf [45]. The program Macclade [47] was used to produce the nexus files for P-Gnome in accordance with the CAOS manual. The most variable sites that distinguish all the taxa were chosen and the character states at these nucleotide positions were listed.

## Results

In total, we analyzed 187 COI (652 bp), 171 16S rDNA (440–530 bp) and 82 ITS-1 (470–560 bp) sequences from 220 *Nassarius* individuals. Sequences from this study were submitted to the GenBank Barcode database with accession numbers JQ975421–JQ975808 listed in Table S1. 40 COI sequences and 12 16S rDNA sequences were obtained from previous studies.

### Phylogenetic, Distance and Character Assignments in COI Barcoding

**Figure 2:**
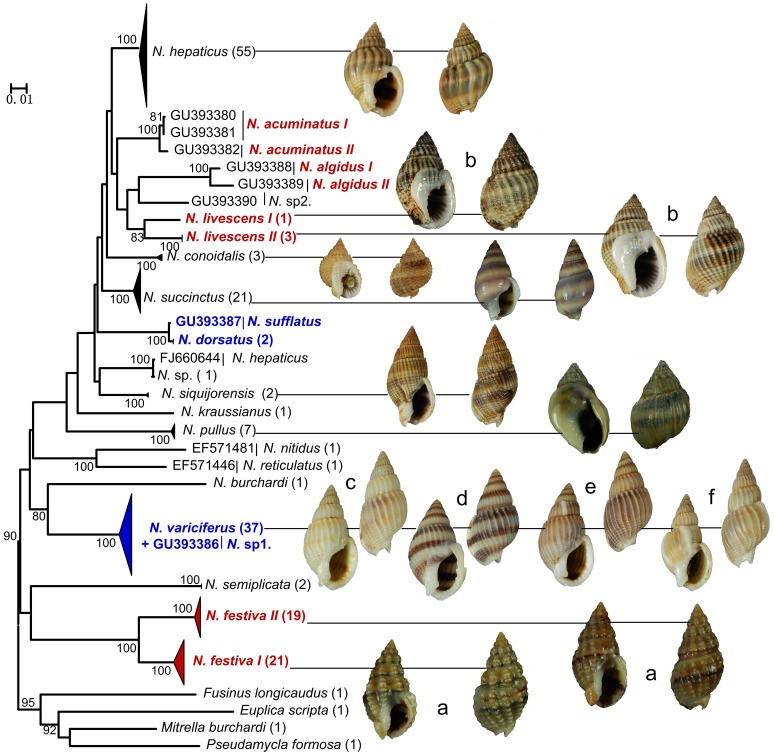
Bayesian tree of the COI locus. Posterior probabilities and bootstrap values were included. The number of individuals included in each species was shown in brackets by the species name. Species showing cryptic diversity were marked in red. Species that could be identified as synonyms were marked in blue. Representative shells of species available were illustrated.

The NJ and Bayesian trees of COI locus supported the monophyly of *Nassarius* (Figure 2). For the 20 *Nassarius* species analyzed, the species *N. hepaticus*, *N. acuminatus*, *N. algidus*, *N. conoidalis*, *N. succinctus*, *N. pullus*, *N. siquijorensis* and *N. semiplicata* formed distinct barcode clusters allowing their unambiguous identification. Two separate clades within *N. festiva* and *N. livescens* were clearly recovered respectively (Figure 2). *N.* sp1 fell within the *N. variciferus* clade. *N.* sp and one individual of *N. hepaticus* (FJ660644) and *N. sufflatus* and *N. dorsatus* were lumped into one lineage respectively.

**Figure 3:**
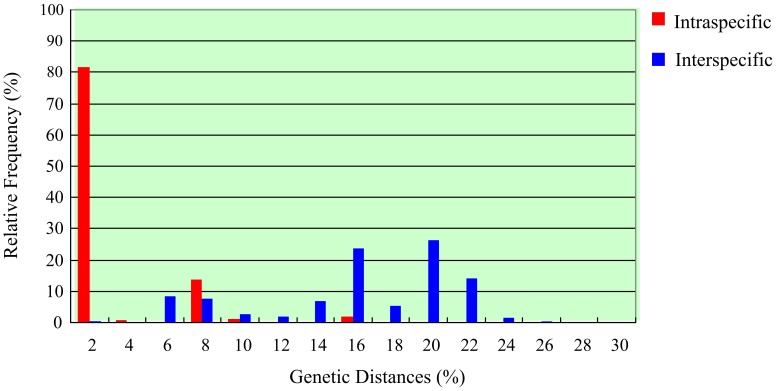
Frequency distribution of COI intraspecific and interspecific (congeneric) K2P distances in *Nassarius*.

The COI pairwise genetic divergences among conspecific individuals ranged from 0% to 16.2% with a mean of 1.19%. Between specimens of different species, the variation was from 0% to 24.80%. The mean interspecific distance was from 0.30% to 22.90% (Table S2). No “distance-gap” was found between intraspecific and interspecific divergences of COI sequences within *Nassarius* (Figure 3). The mean distances between two clades within *N. festiva* and *N. livescens* were 6.00% and 4.5% respectively. The mean genetic divergences between *N.* sp1 and *N. variciferus* and *N. sufflatus* and *N. dorsatus* were only 0.60% and 0.30% respectively.

The COI NJ tree was the guide tree for COI CAOS analysis. 22 defined clades in Figure 2 were analyzed: *N. festiva I*, *N. festiva II, N. hepaticus, N. succinctus, N. siquijorensis, N. dorsatus, N. pullus, N. semiplicata, N. conoidalis, N. livescens I, N. livescens II, N.* sp (including FJ6606441), *N. variciferus, N. acuminatus, N*. sp1, *N. algidus, N. sufflatus, N. burchardi, N. kraussianus, N. nitidus, N. reticulates* and *N.* sp2. In the COI gene region of 22 clades character states at 41 nucleotide positions were detected (Table S3). The particular nucleotide positions were chosen due to the high number of CAs at the important nodes or because of the presence of CAs for groups with highly similar sequences. All the clades except *N. variciferus*, *N.* sp1, *N. dorsatus* and *N. sufflatus* revealed a unique combination of character states at 41 nucleotide positions with at least 3 CAs for each. *N. festiva I* and *N. festiva II* and *N. livescens I* and *N. livescens II* were clearly separated respectively with more than 8 CAs. Two separate clades within *N. acuminatus* and *N. algidus* in COI phylogenetic tree (see Figure 2) were also detected with 3 and 5 CAs respectively.

### Phylogenetic and Character Assignments in 16S rDNA Barcoding

**Figure 4:**
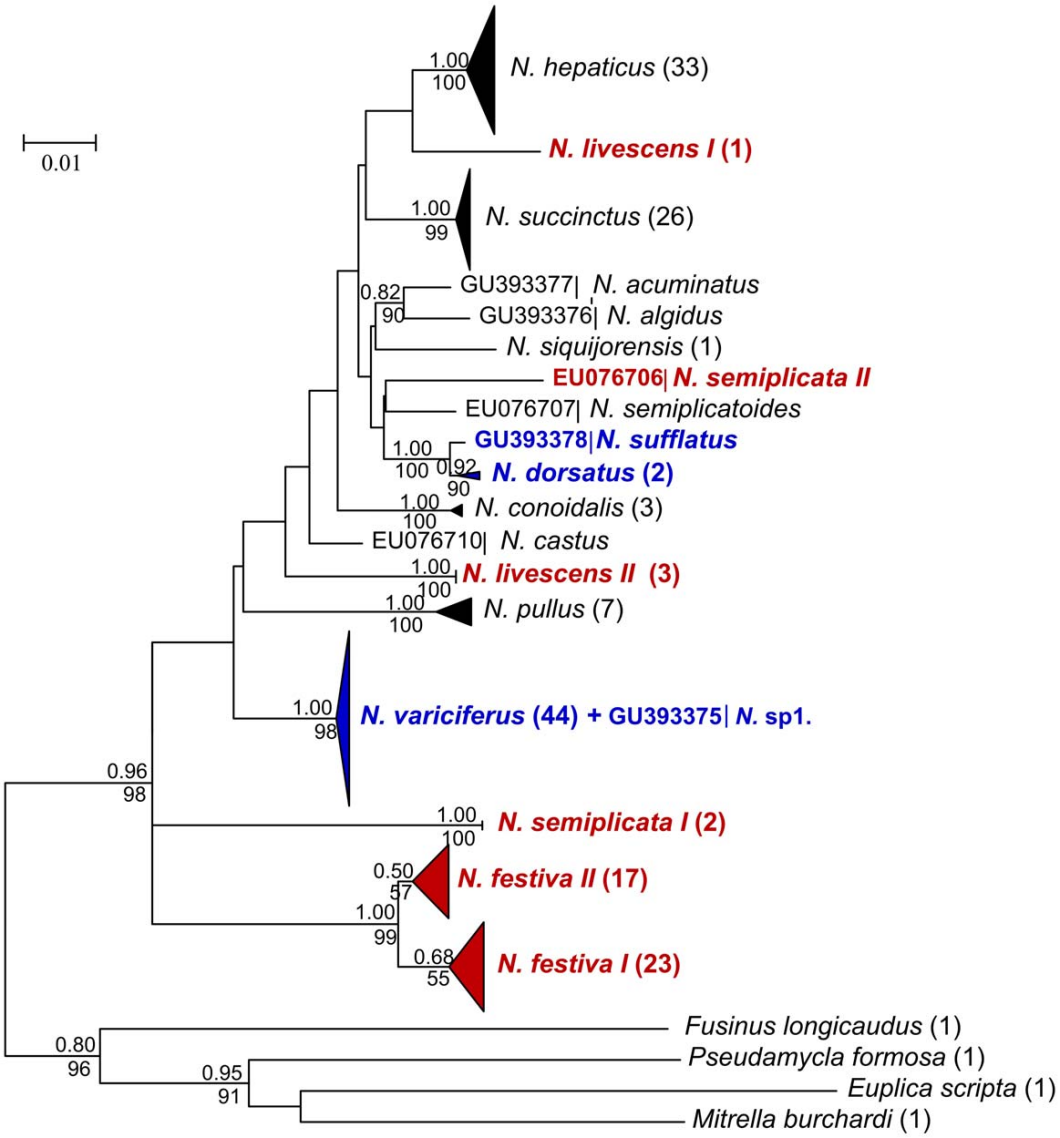
Bayesian tree of the 16S rDNA locus. Posterior probabilities and bootstrap values were included. The number of individuals included in each species was shown in brackets by the species name. Species showing cryptic diversity were marked in red. Species that could be identified as synonyms were marked in blue.

Generally, the 16S rDNA NJ and Bayesian trees revealed same resolution to COI trees (Figure 4). For the 16 *Nassarius* species analyzed, the monophyly of *N. hepaticus*, *N. succinctus*, *N. conoidalis*, *N. pullus* were strongly supported. Although with weak support, *N. festiva* was separated into two clades (Figure 4). *N. livescens* was also clearly separated into two clusters. *N. variciferus* and *N.* sp1 and *N. sufflatus* and *N. dorsatu* grouped together with each other respectively. Unexpectedly, one individual of *N. semiplicata* (EU076706) failed to group together with other individuals.

The 16S rDNA NJ tree was the guide tree for 16S rDNA CAOS analysis. 19 defined clades in Figure 4 were analyzed: *N. festiva I*, *N. festiva II*, *N. hepaticus*, *N. succinctus*, *N. siquijorensis*, *N. dorsatus*, *N. pullus*, *N. semiplicata I*, *N. semiplicata II*, *N. conoidalis*, *N. livescens I*, *N. livescens II*, *N. variciferus*, *N.* sp1, *N. sufflatus*, *N. semiplicatoides*, *N. acuminatus*, *N. algidus* and *N. castus*. In the 16S rDNA gene region of 19 clades character states at 30 nucleotide positions were detected (Table S4). All the clades except *N. variciferus*, *N*. sp1, *N. dorsatus* and *N. sufflatus* revealed a unique combination of character states at 30 nucleotide positions with at least 3 CAs for each. *N. festiva I* and *N. festiva II* and *N. livescens I* and *N. livescens II* were clearly separated respectively with more than 5 CAs. For *N. semiplicata I* and *N. semiplicata II*, 20 CAs were found.

### Phylogenetic and Character Assignments in ITS-1 Barcoding

**Figure 5:**
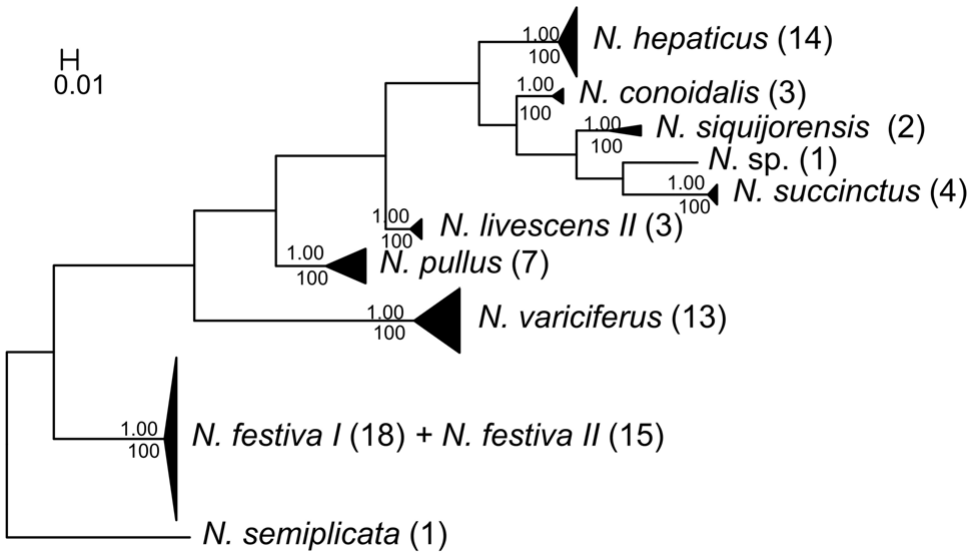
Bayesian tree of the ITS-1 locus. Posterior probabilities and bootstrap values were included. The number of individuals included in each species was shown in brackets by the species name.

For the 10 *Nassarius* species analyzed, 10 distinct *Nassarius* lineages can be identified in ITS-1 NJ and Bayesian trees (Figure 5). The species *N. hepaticus*, *N. siquijorensis*, *N. succinctus*, *N. conoidalis*, *N. pullus*, *N. livescens* and *N. variciferus* were recovered as monophyletic. However, the ITS-1 region failed to separate *N. festiva I* and *N. festiva II* recovered in COI and 16S rDNA trees.

The ITS-1 NJ tree was the guide tree for ITS-1 CAOS analysis. 11 clades in Figure 5 were analyzed: *N. festiva I*, *N. festiva II*, *N. hepaticus*, *N. succinctus*, *N. siquijorensis*, *N. pullus*, *N.* sp, *N. semiplicata*, *N. conoidalis*, *N. livescens* and *N. variciferus*. In the ITS-1 gene region of 11 clades character states at 26 nucleotide positions were found (Table S5). All the clades except *N. festiva I* and *N. festiva II* revealed a unique combination of character states at 26 nucleotide positions with at least 3 CAs for each.

## Discussion

### Species Delimitation and Cryptic Diversity

DNA sequence data now offers an effective tool for taxonomic studies by greatly expanding the number of characters that can be used to distinguish species. The inclusion of such data, along with the traditional morphological variables, promises to rectify the problem of subjectivity in current species descriptions [48], [49], [50]. Our analyses of comprehensive samples of *Nassarius* species, combining genetic data with morphological characters (discussed below), led to the successful identification of 12 *Nassarius* species and the discovery of four cryptic species, one pair synonyms and one intraspecific morphologically diverse species.

First, the genetic data and the morphological characters provided the most obvious evidence for the existence of one cryptic species in *N. festiva*. In this study, all the individuals of *N. festiva* were separated into two different lineages (*N. festiva I* and *N. festiva II*) in COI and 16S rDNA phylogenetic trees. The two lineages were also clearly recovered in COI and 16S rDNA character assignments with many CAs. Moreover, the COI divergence between *N. festiva I* and *N. festiva II* was larger than the mean intraspecific divergence. However, the ITS-1 region failed to separate the two clades. The reason may be that ITS-1 gene does not have sufficient variation to distinguish the two recent diverged lineages since it evolve much more slowly than COI and 16S rDNA genes [51], [52]. Slightly ambiguous, but still significant different morphological trait between *N. festiva I* and *N. festiva II* is that the verrucous protuberances on the shell of *N. festiva I* are bigger than that on the shell of *N. festiva II* (Figure 2 (*a*)). Geographily, the two clades are both represented in the same localities in this study. Wang *et al.* [53] suggested that *N. dealbatus* be the synonym of *N. festiva*. Here our study suggests that *N. festiva* be regarded as two separate species.

Second, both phylogenetic trees and character assignments of COI and 16S rDNA genes separated one individual of *N. livescens* (*N. livescens I*) from other individuals (*N. livescens II*). The mean COI distance between *N. livescens I* and *N. livescens II* was also larger than the mean intraspecific divergence. All the individuals of the two clades were collected from the same localities in this study. Specimens of the two clades are almost identical morphologically but a putative difference may be that there are more axial ribs on the shell of *N. livescens II* than that on the shell of *N. livescens I* (Figure 2 (*b*)). Thus, a putative cryptic species within *N. livescens* is found and more individuals from more localities are needed to find more morphological and genetic differences between the two clades.

Third, the species *N. acuminatus* and *N. algidus* also showed cryptic genetic diversity. Although all the individuals of *N. acuminatus* and *N. algidus* fell into one cluster respectively in COI phylogenetic trees, two clades within each species in COI trees were clearly separated in COI character assignments. The cryptic diversity within the two species needs to be recognized.

Fourth, although all analysis of COI, 16S rDNA and ITS-1 sequences supported the monophyletic of *N. variciferus*, *N. variciferus* showed high intraspecific morphological diversity. First of all, it should be noted that some individuals of *N. variciferus* have no varices on the shell (Figure 2 (*c*) and (*d*)). Some individuals just have varices on body whorl or one spiral whorl (Figure 2 (*e*) and (*f*)). Thus, it is wrong that all individuals of *N. variciferus* have distinct varices on body whorl and all spiral whorls. In addition, the color of spiral bands of some individuals is much darker than that of other individuals (Figure 2 (*d*)) and the suture of some individuals is a little deeper than that of others (Figure 2 (*f*)). Therefore, we must be cautious to identify the specimens of *N. variciferus* since there is high morphological diversity within it. The unknown species *N*. sp1 (GU393386 in Li *et al.* [38]) also fell into the *N. variciferus* clade in both COI and 16S rDNA phylogenetic trees, and same CAs were detected for them in both COI and 16S rDNA character assignments. Therefore, *N*. sp1 can be identified as *N. variciferus*.

Finally, *N. sufflatus* grouped together with *N. dorsatus* with 99% or 100% support in COI and 16S rDNA phylogenetic trees. Same CAs were also detected for *N. sufflatus* and *N. dorsatus* in both COI and 16S rDNA character assignments, and the mean COI distance between them was only o.3%. Thus, *N. sufflatus* and *N. dorsatus* could be regarded as synonyms. Unexpectedly, one individual of *N. hepaticus* (FJ6606441 in Wang *et al.* [53]) was separated from all others and grouped together with *N.* sp in both COI phylogenetic and character analysis. The individual may be misidentified by Wang *et al.* [53]. Another unknown species *N.* sp2 (GU393390 in Li *et al*. [38]) was not identified. It also failed to be identified to species level in the Barcode of Life Data Systems (BOLD). Thus, more sequences need to be produced in BOLD for the identification of unknown species. The other nominal species, e.g. *N. hepaticus*, *N. succinctus*, *N. pullus*, *N. semiplicata*, *N. conoidalis* and *N. siquijorensis* examined in this study, were successfully identified in phylogenetic trees and character assignments of COI, 16S rDNA and ITS-1 sequences. In these species clades, no geographical clusters could be detected.

### DNA Barcoding and Three Methods

This study has shown that DNA barcoding is effective in identifying *Nassarius* species. It can reveal cryptic species that morphological characters can not distinguish alone due to the intraspecific variation and various intraspecific forms. The correct identification and revelation of cryptic diversity is important for *Nassarius* in species conversation, food safety and better understanding the patterns of ecosystem functioning. Actually, like *Nassarius*, the external morphology of most marine species is easily affected by the environmental factors, at least for the mollusk, which makes morphological characters sometimes unreliable to identify. Thus, DNA barcoding will be a powerful tool for revealing the marine biodiversity. At the fewest, DNA barcoding can flag species and educe the candidate new species, after which the traditional characters can complement the identification. Whatever, the integration of distinct DNA characters and traditional information such as morphology and geography in a comprehensive character-based barcode database is needed for fast species identification and discovery.

In this case study, the traditional barcoding methods, monophyly and distance-based methods, were very helpful in revealing the diversity of *Nassarius* species. For example, all phylogenetic trees of COI, 16S rDNA and ITS-1 genes could recover most species (including the cryptic species) as monophyletic and the COI interspecific divergences were generally higher than the intraspecific divergences. Even so, compared with the character-based DNA barcoding, some limits of the traditional barcoding methods still appeared. First, identification does not hinge on monophyly and the use of reciprocal monophyly as a criterion for species recognition is arbitrary [54], [55]. In this study, although some species were recovered as monophyletic in the phylogenetic trees, the cryptic diversity within the species could not be completely shown in the trees. For example, within the monophyletic species *N. festiva* in 16S rDNA trees, the clades *N. festiva I* and *N. festiva II* were weakly supported (see Figure 4), but they were clearly separated in 16S rDNA character assignments (Table S4). In addition, two closely related clades within *N. acuminatus* and *N. algidus* in COI trees (see Figure 2) were also detected respectively in COI character assignments (Table S3). Moreover, if one species is represented with a single individual in phylogenetic profile, it is not determinative the species is monophyletic or polyphyletic (e.g. *N. sufflatus* and *N. dorsatus* and *N. nitidus* and *N. reticulatus* in COI phylogenetic trees). Nevertheless, a character-based DNA barcode of a single individual is still useful and provides important information for this species within a group of interest. Second, the distance-based approach failed in some species in this study. No “barcoding gap” was found between COI intra- and interspecific variation. On the contrary, there was obvious overlap between them. In addition, since some cryptic species existed the “10× rule” threshold (11.9% in this study) proposed by Hebert *et al.* [52] was too liberal to recognize some distinct species. The character-based method of DNA barcoding, however, was effective for the identification of genetic entities. It could easily detect the cryptic species that could not be recovered with NJ profile and genetic distance and the species that were represented by a single individual. Although there is no absolute certainty for a given CA to be fixed, the reliability of a barcode increases with each additional independent CA added [24]. Another advantage of character-based barcoding is the fact that it is compatible with classical approaches allowing the combination of classical morphological information.

### Food Safety in *Nassarius*

Nassariids of *Nassarius* are popular with people in China since they are very delicious to eat. However, it is dangerous to consume them as food since different toxins are concentrated in *Nassarius* sp’s body. Food poisoning incidents caused by eating nassariids of *Nassarius* have been reported frequently in the last several years in China. Many people died of the poisoning incidents [31], [32]. Thus, relevant departments of China have forbidden selling nassariids with toxins. The origin of the toxicity in *Nassarius* sp’s body is still unclear. It is inferred that the toxicity probably originates from the food chains, actinomycetes in *Nassarius* sp’s body or an enzyme produced by themselves. Some studies find that the toxicity of *Nassarius* is relative to species [31], [32]. While some species are toxic and some species are non-toxic, the toxicity of some species changes with the season [31], [32]. Therefore, correct species identification is the basis of studying *Nassarius* toxicity. However, the morphological confusion in *Nassarius* often results in error in virulence judgment. Here our comprehensive barcoding study for species delimitation and cryptic diversity revelation of *Nassarius* will greatly contribute to the virulence study of *Nassarius* since representatives of toxic, non-toxic and season-toxic species are all included in our study.

## Supporting Information

Table S1 **Sampling of** ***Nassarius*** **species and outgroups studied.**
(DOC)

Table S2 **The mean interspecific divergences of COI sequences.**
(DOC)

Table S3 **Character-based DNA barcodes for COI gene.**
(DOC)

Table S4 **Character-based DNA barcodes for 16S rDNA gene.**
(DOC)

Table S5 **Character-based DNA barcodes for ITS-1 gene.**
(DOC)
