# Supplementary material for: Monophyly, Distance and Character–Based Multigene Barcoding Reveal Extraordinary Cryptic Diversity in Nassarius: A Complex and Dangerous Community
Source: PLoS One. 2012 Oct 11;7(10):e47276. doi: 10.1371/journal.pone.0047276 (PMC3469534; doi:10.1371/journal.pone.0047276)
Supplement: Table S1 — Sampling of Nassarius species and outgroups studied. (DOC) [file pone.0047276.s001.doc]

Table S1. Voucher number, locality and GenBank numbers of the studied *Nassarius* specimens and outgroups. Site codes correspond to geographic locations listed in Figure 1. Samples in bold were previously published.

| Species | Museum voucher no. | Locality | Site code | COI | 16S rDNA | ITS-1 |
| --- | --- | --- | --- | --- | --- | --- |
| *N. festiva* |  |  |  |  |  |  |
|  | LSGB23401B1 | Jimo, Shandong, China | JS | JQ975438 | JQ975571 | JQ975730 |
|  | LSGB23401B3 | Jimo, Shandong, China | JS | JQ975422 | JQ975572 | –– |
|  | LSGB23401B4 | Jimo, Shandong, China | JS | JQ975573 | JQ975573 | JQ975731 |
|  | LSGB23401B5 | Jimo, Shandong, China | JS | –– | JQ975579 | –– |
|  | LSGB23401B6 | Jimo, Shandong, China | JS | JQ975443 | JQ975574 | JQ975732 |
|  | LSGB23401B7 | Jimo, Shandong, China | JS | JQ975444 | JQ975575 | JQ975733 |
|  | LSGB23401B8 | Jimo, Shandong, China | JS | JQ975423 | JQ975576 | JQ975734 |
|  | LSGB23401B9 | Jimo, Shandong, China | JS | –– | JQ975580 | –– |
|  | LSGB23401B10 | Jimo, Shandong, China | JS | JQ975445 | –– | –– |
|  | LSGB23401B11 | Jimo, Shandong, China | JS | JQ975446 | JQ975577 | JQ975735 |
|  | LSGB23401B12 | Jimo, Shandong, China | JS | JQ975447 | JQ975578 | –– |
|  | LSGB23401D1 | Kenli, Shandong, China | KS | JQ975449 | JQ975584 | –– |
|  | LSGB23401D2 | Kenli, Shandong, China | KS | JQ975450 | JQ975585 | JQ975739 |
|  | LSGB23401D3 | Kenli, Shandong, China | KS | –– | JQ975586 | –– |
|  | LSGB23401D4 | Kenli, Shandong, China | KS | JQ975452 | –– | JQ975740 |
|  | LSGB23401D5 | Kenli, Shandong, China | KS | JQ975453 | JQ975587 | JQ975741 |
|  | LSGB23401D6 | Kenli, Shandong, China | KS | JQ975454 | –– | JQ975742 |
|  | LSGB23401D7 | Kenli, Shandong, China | KS | JQ975455 | JQ975588 | JQ975743 |
|  | LSGB23401D8 | Kenli, Shandong, China | KS | JQ975456 | JQ975589 | –– |
|  | LSGB23401D9 | Kenli, Shandong, China | KS | –– | JQ975593 | –– |
|  | LSGB23401D10 | Kenli, Shandong, China | KS | JQ975457 | JQ975590 | JQ975744 |
|  | LSGB23401D11 | Kenli, Shandong, China | KS | JQ975458 | JQ975591 | JQ975745 |
|  | LSGB23401D12 | Kenli, Shandong, China | KS | JQ975459 | JQ975592 | JQ975746 |
|  | LSGB23401F1 | Ningbo, Zhejiang, China | NZ | JQ975436 | JQ975602 | JQ975753 |
|  | LSGB23401F2 | Ningbo, Zhejiang, China | NZ | JQ975427 | JQ975603 | JQ975754 |
|  | LSGB23401F3 | Ningbo, Zhejiang, China | NZ | JQ975424 | JQ975604 | JQ975755 |
|  | LSGB23401F4 | Ningbo, Zhejiang, China | NZ | JQ975461 | –– | –– |
|  | LSGB23401F5 | Ningbo, Zhejiang, China | NZ | JQ975441 | JQ975605 | JQ975756 |
|  | LSGB23401F6 | Ningbo, Zhejiang, China | NZ | JQ975428 | JQ975606 | JQ975757 |
|  | LSGB23401F7 | Ningbo, Zhejiang, China | NZ | JQ975437 | JQ975607 | JQ975758 |
|  | LSGB23401F8 | Ningbo, Zhejiang, China | NZ | JQ975435 | JQ975608 | JQ975759 |
|  | LSGB23401E1 | Caotan, Guangdong, China | CG | JQ975421 | JQ975594 | JQ975747 |
|  | LSGB23401E2 | Caotan, Guangdong, China | CG | JQ975429 | JQ975595 | –– |
|  | LSGB23401E3 | Caotan, Guangdong, China | CG | JQ975426 | JQ975596 | JQ975748 |
|  | LSGB23401E4 | Caotan, Guangdong, China | CG | JQ975430 | JQ975597 | JQ975749 |
|  | LSGB23401E5 | Caotan, Guangdong, China | CG | JQ975460 | JQ975598 | JQ975750 |
|  | LSGB23401E6 | Caotan, Guangdong, China | CG | JQ975440 | JQ975599 | JQ975751 |
|  | LSGB23401E7 | Caotan, Guangdong, China | CG | JQ975434 | JQ975600 | JQ975752 |
|  | LSGB23401E8 | Caotan, Guangdong, China | CG | –– | JQ975601 | –– |
|  | LSGB23401A1 | Jiaowei, Guangdong, China | JG | JQ975431 | JQ975568 | JQ975727 |
|  | LSGB23401A2 | Jiaowei, Guangdong, China | JG | JQ975433 | JQ975569 | JQ975728 |
|  | LSGB23401A3 | Jiaowei, Guangdong, China | JG | JQ975425 | JQ975570 | JQ975729 |
|  | LSGB23401C1 | Xinliao, Guangdong, China | XG | JQ975448 | JQ975581 | JQ975736 |
|  | LSGB23401C2 | Xinliao, Guangdong, China | XG | JQ975432 | JQ975582 | JQ975737 |
|  | LSGB23401C3 | Xinliao, Guangdong, China | XG | JQ975439 | JQ975583 | JQ975738 |
| *N. hepaticus* |  |  |  |  |  |  |
|  | LSGB23403A1 | Beihai, Guangxi, China | BG | JQ975462 | JQ975609 | JQ975760 |
|  | LSGB23403A2 | Beihai, Guangxi, China | BG | JQ975463 | JQ975610 | JQ975761 |
|  | LSGB23403A3 | Beihai, Guangxi, China | BG | JQ975464 | JQ975611 | –– |
|  | LSGB23403A5 | Beihai, Guangxi, China | BG | JQ975465 | –– | JQ975762 |
|  | LSGB23403A6 | Beihai, Guangxi, China | BG | JQ975466 | JQ975612 | –– |
|  | LSGB23403A7 | Beihai, Guangxi, China | BG | JQ975467 | JQ975613 | JQ975763 |
|  | LSGB23403A10 | Beihai, Guangxi, China | BG | JQ975468 | JQ975614 | –– |
|  | LSGB23403A11 | Beihai, Guangxi, China | BG | JQ975469 | JQ975615 | JQ975764 |
|  | LSGB23403A12 | Beihai, Guangxi, China | BG | JQ975470 | JQ975616 | –– |
|  | LSGB23403A13 | Beihai, Guangxi, China | BG | JQ975471 | JQ975619 | –– |
|  | LSGB23403A14 | Beihai, Guangxi, China | BG | JQ975472 | JQ975617 | –– |
|  | LSGB23403A15 | Beihai, Guangxi, China | BG | JQ975473 | JQ975618 | –– |
|  | LSGB23403A16 | Beihai, Guangxi, China | BG | JQ975474 | JQ975620 | –– |
|  | LSGB23403A17 | Beihai, Guangxi, China | BG | JQ975475 | JQ975622 | –– |
|  | LSGB23403A22 | Beihai, Guangxi, China | BG | –– | JQ975621 | JQ975766 |
|  | LSGB23403A21 | Beihai, Guangxi, China | BG | –– | –– | JQ975765 |
|  | LSGB23403B1 | Zhanjiang, Guangdong, China | ZG | JQ975476 | JQ975623 | –– |
|  | LSGB23403B2 | Zhanjiang, Guangdong, China | ZG | JQ975477 | JQ975624 | –– |
|  | LSGB23403B3 | Zhanjiang, Guangdong, China | ZG | JQ975478 | JQ975625 | –– |
|  | LSGB23403B4 | Zhanjiang, Guangdong, China | ZG | JQ975479 | JQ975626 | –– |
|  | LSGB23403B5 | Zhanjiang, Guangdong, China | ZG | JQ975480 | JQ975627 | JQ975767 |
|  | LSGB23403B7 | Zhanjiang, Guangdong, China | ZG | JQ975481 | JQ975631 | –– |
|  | LSGB23403B9 | Zhanjiang, Guangdong, China | ZG | JQ975482 | JQ975630 | –– |
|  | LSGB23403B10 | Zhanjiang, Guangdong, China | ZG | JQ975483 | JQ975632 | –– |
|  | LSGB23403B11 | Zhanjiang, Guangdong, China | ZG | –– | JQ975633 | JQ975768 |
|  | LSGB23403B12 | Zhanjiang, Guangdong, China | ZG | JQ975484 | JQ975634 | JQ975769 |
|  | LSGB23403B13 | Zhanjiang, Guangdong, China | ZG | JQ975485 | JQ975635 | –– |
|  | LSGB23403B14 | Zhanjiang, Guangdong, China | ZG | –– | JQ975628 | –– |
|  | LSGB23403B15 | Zhanjiang, Guangdong, China | ZG | –– | –– | JQ975770 |
|  | LSGB23403B16 | Zhanjiang, Guangdong, China | ZG | JQ975486 | JQ975636 | –– |
|  | LSGB23403B17 | Zhanjiang, Guangdong, China | ZG | JQ975487 | JQ975629 | JQ975771 |
|  | LSGB23403B20 | Zhanjiang, Guangdong, China | ZG | JQ975488 | –– | –– |
|  | LSGB23403B21 | Zhanjiang, Guangdong, China | ZG | JQ975489 | JQ975641 | JQ975772 |
|  | LSGB23403B22 | Zhanjiang, Guangdong, China | ZG | JQ975490 | JQ975640 | –– |
|  | LSGB23403B23 | Zhanjiang, Guangdong, China | ZG | JQ975491 | JQ975637 | –– |
|  | LSGB23403B25 | Zhanjiang, Guangdong, China | ZG | JQ975492 | JQ975639 | –– |
|  | **?** | **Fujian, China** | **FG** | **FJ660643-FJ660667** | –– | –– |
| *N. succinctus* |  |  |  |  |  |  |
|  | LSGB23405B1 | Rizhao, Shandong, China | RS | JQ975507 | JQ975659 | JQ975775 |
|  | LSGB23405B2 | Rizhao, Shandong, China | RS | JQ975508 | JQ975660 | –– |
|  | LSGB23405B6 | Rizhao, Shandong, China | RS | JQ975509 | JQ975661 | –– |
|  | LSGB23405B8 | Rizhao, Shandong, China | RS | –– | –– | JQ975776 |
|  | LSGB23405B10 | Rizhao, Shandong, China | RS | –– | JQ975665 | JQ975777 |
|  | LSGB23405B11 | Rizhao, Shandong, China | RS | JQ975510 | JQ975664 | –– |
|  | LSGB23405B15 | Rizhao, Shandong, China | RS | JQ975511 | JQ975666 | –– |
|  | LSGB23405B19 | Rizhao, Shandong, China | RS | JQ975512 | JQ975663 | –– |
|  | LSGB23405B22 | Rizhao, Shandong, China | RS | JQ975513 | JQ975662 | –– |
|  | LSGB23405A1 | Beihai, Guangxi, China | BG | –– | JQ975658 | –– |
|  | LSGB23405A2 | Beihai, Guangxi, China | BG | JQ975504 | JQ975642 | –– |
|  | LSGB23405A3 | Beihai, Guangxi, China | BG | –– | JQ975643 | –– |
|  | LSGB23405A4 | Beihai, Guangxi, China | BG | JQ975495 | JQ975647 | –– |
|  | LSGB23405A5 | Beihai, Guangxi, China | BG | JQ975496 | JQ975646 | –– |
|  | LSGB23405A6 | Beihai, Guangxi, China | BG | JQ975497 | JQ975645 | –– |
|  | LSGB23405A7 | Beihai, Guangxi, China | BG | JQ975499 | JQ975649 | –– |
|  | LSGB23405A8 | Beihai, Guangxi, China | BG | JQ975498 | JQ975644 | JQ975773 |
|  | LSGB23405A10 | Beihai, Guangxi, China | BG | JQ975494 | JQ975650 | –– |
|  | LSGB23405A11 | Beihai, Guangxi, China | BG | JQ975505 | JQ975651 | JQ975774 |
|  | LSGB23405A12 | Beihai, Guangxi, China | BG | JQ975500 | JQ975652 | –– |
|  | LSGB23405A13 | Beihai, Guangxi, China | BG | JQ975501 | JQ975648 | –– |
|  | LSGB23405A19 | Beihai, Guangxi, China | BG | –– | JQ975653 | –– |
|  | LSGB23405A20 | Beihai, Guangxi, China | BG | JQ975503 | JQ975654 | –– |
|  | LSGB23405A21 | Beihai, Guangxi, China | BG | JQ975506 | JQ975655 | –– |
|  | LSGB23405A22 | Beihai, Guangxi, China | BG | –– | JQ975656 | –– |
|  | LSGB23405A23 | Beihai, Guangxi, China | BG |  | JQ975657 |  |
|  | **?** | **?** | **?** | –– | **EU502711** | –– |
| *N. siquijorensis* |  |  |  |  |  |  |
|  | LSGB23407D1 | Zhanjiang, Guangdong, China | ZG | –– | –– | JQ975778 |
|  | LSGB23407D2 | Zhanjiang, Guangdong, China | ZG | JQ975552 | JQ975667 | JQ975779 |
|  | **?** | **?** | **?** | **EU124789** | –– | –– |
| *N. dorsatus* |  |  |  |  |  |  |
|  | LSGB23409A1 | Zhanjiang, Guangdong, China | ZG | JQ975553 | JQ975713 | –– |
|  | LSGB23409A2 | Zhanjiang, Guangdong, China | ZG | JQ975554 | JQ975714 | –– |
| *N. pullus* |  |  |  |  |  |  |
|  | LSGB23406A1 | Beihai, Guangxi, China | BG | JQ975555 | JQ975715 | JQ975797 |
|  | LSGB23406A2 | Beihai, Guangxi, China | BG | JQ975556 | JQ975716 | JQ975798 |
|  | LSGB23406A3 | Beihai, Guangxi, China | BG | JQ975557 | JQ975717 | JQ975799 |
|  | LSGB23406A4 | Beihai, Guangxi, China | BG | JQ975558 | JQ975718 | JQ975800 |
|  | LSGB23406A5 | Beihai, Guangxi, China | BG | JQ975559 | JQ975719 | –– |
|  | LSGB23406B1 | Xinliao, Guangdong, China | XG | JQ975560 | JQ975720 | JQ975802 |
|  | LSGB23406C1 | Caotan, Guangdong, China | CG | JQ975561 | JQ975721 | JQ975803 |
| *N. semiplicata* |  |  |  |  |  |  |
|  | LSGB23408B1 | Xinliao, Guangdong, China | XG | JQ975563 | JQ975722 | JQ975805 |
|  | LSGB23408B2 | Xinliao, Guangdong, China | XG | JQ975564 | JQ975723 | –– |
|  | **?** | **?** | **?** | –– | **EU076706** | –– |
| *N. conoidalis* |  |  |  |  |  |  |
|  | LSGB23402A2 | Zhanjiang, Guangdong, China | ZG | JQ975565 | JQ975724 | JQ975806 |
|  | LSGB23402A3 | Zhanjiang, Guangdong, China | ZG | JQ975566 | JQ975725 | JQ975807 |
|  | LSGB23402A4 | Zhanjiang, Guangdong, China | ZG | JQ975567 | JQ975726 | JQ975808 |
| *N. livescens* |  |  |  |  |  |  |
|  | LSGB234010A1 | Wenchang, Hainan, China | WH | JQ975514 | JQ975670 | JQ975780 |
|  | LSGB234010A2 | Wenchang, Hainan, China | WH | JQ975515 | –– | JQ975781 |
|  | LSGB234010A3 | Wenchang, Hainan, China | WH | JQ975516 | –– | JQ975782 |
|  | LSGB234010A5 | Wenchang, Hainan, China | WH | JQ975517 | JQ975671 | JQ975783 |
| *N.* sp | LSGB2340117A1 | Beihai, Guangxi, China | BG | JQ975562 | –– | JQ975804 |
| *N. variciferus* |  |  |  |  |  |  |
|  | LSGB23404E1 | Ningbo, Zhejiang, China | NZ | JQ975537 | JQ975696 | –– |
|  | LSGB23404E2 | Ningbo, Zhejiang, China | NZ | JQ975538 | JQ975697 | JQ975789 |
|  | LSGB23404E3 | Ningbo, Zhejiang, China | NZ | JQ975539 | JQ975698 | JQ975790 |
|  | LSGB23404E4 | Ningbo, Zhejiang, China | NZ | JQ975540 | JQ975699 | –– |
|  | LSGB23404E5 | Ningbo, Zhejiang, China | NZ | JQ975541 | JQ975700 | JQ975791 |
|  | LSGB23404E6 | Ningbo, Zhejiang, China | NZ | JQ975542 | JQ975701 | –– |
|  | LSGB23404E7 | Ningbo, Zhejiang, China | NZ | JQ975543 | JQ975702 | –– |
|  | LSGB23404E8 | Ningbo, Zhejiang, China | NZ | JQ975544 | JQ975703 | –– |
|  | LSGB234049A1 | Ningbo, Zhejiang, China | NZ | JQ975545 | JQ975706 | JQ975796 |
|  | LSGB234049A2 | Ningbo, Zhejiang, China | NZ | JQ975546 | JQ975707 | JQ975794 |
|  | LSGB234049A3 | Ningbo, Zhejiang, China | NZ | JQ975547 | JQ975708 | –– |
|  | LSGB234049A4 | Ningbo, Zhejiang, China | NZ | JQ975548 | JQ975709 | JQ975795 |
|  | LSGB234049A5 | Ningbo, Zhejiang, China | NZ | JQ975549 | JQ975710 | –– |
|  | LSGB234049A6 | Ningbo, Zhejiang, China | NZ | JQ975550 | JQ975711 | –– |
|  | LSGB234049A7 | Ningbo, Zhejiang, China | NZ | JQ975551 | JQ975712 | –– |
|  | LSGB23404E9 | Ningbo, Zhejiang, China | NZ | –– | JQ975704 | JQ975792 |
|  | LSGB23404E10 | Ningbo, Zhejiang, China | NZ | –– | JQ975705 | JQ975793 |
|  | LSGB23404B2 | Panjin, Liaoning, China | PL | JQ975518 | JQ975674 | JQ975784 |
|  | LSGB23404B4 | Panjin, Liaoning, China | PL | JQ975519 | JQ975673 | –– |
|  | LSGB23404B6 | Panjin, Liaoning, China | PL | JQ975520 | JQ975672 | –– |
|  | LSGB23404B9 | Panjin, Liaoning, China | PL | JQ975521 | –– | –– |
|  | LSGB23404B10 | Panjin, Liaoning, China | PL | JQ975522 | JQ975675 | –– |
|  | LSGB23404B11 | Panjin, Liaoning, China | PL | JQ975523 | JQ975676 | –– |
|  | LSGB23404B12 | Panjin, Liaoning, China | PL | JQ975524 | JQ975677 | JQ975786 |
|  | LSGB23404B13 | Panjin, Liaoning, China | PL | JQ975525 | JQ975678 | –– |
|  | LSGB23404B14 | Panjin, Liaoning, China | PL | JQ975526 | JQ975679 | –– |
|  | LSGB23404B15 | Panjin, Liaoning, China | PL | JQ975527 | JQ975680 | JQ975787 |
|  | LSGB23404B16 | Panjin, Liaoning, China | PL | JQ975528 | JQ975681 | –– |
|  | LSGB23404B18 | Panjin, Liaoning, China | PL | JQ975529 | JQ975682 | –– |
|  | LSGB23404B19 | Panjin, Liaoning, China | PL | JQ975530 | JQ975683 | –– |
|  | LSGB23404B21 | Panjin, Liaoning, China | PL | JQ975531 | JQ975685 | –– |
|  | LSGB23404B17 | Panjin, Liaoning, China | PL | –– | JQ975687 | –– |
|  | LSGB23404B20 | Panjin, Liaoning, China | PL | –– | –– | –– |
|  | LSGB23404B23 | Panjin, Liaoning, China | PL | –– | JQ975686 | –– |
|  | LSGB23404B8 | Panjin, Liaoning, China | PL | –– | –– | JQ975785 |
|  | LSGB23404C2 | Ganyu, Jiangsu, China | GJ | JQ975532 | JQ975689 | –– |
|  | LSGB23404C4 | Ganyu, Jiangsu, China | GJ | JQ975533 | JQ975690 | –– |
|  | LSGB23404C5 | Ganyu, Jiangsu, China | GJ | JQ975534 | JQ975691 | –– |
|  | LSGB23404C6 | Ganyu, Jiangsu, China | GJ | JQ975535 | JQ975692 | –– |
|  | LSGB23404C12 | Ganyu, Jiangsu, China | GJ | JQ975536 | –– | –– |
|  | LSGB23404C1 | Ganyu, Jiangsu, China | GJ | –– | JQ975688 | –– |
|  | LSGB23404C7 | Ganyu, Jiangsu, China | GJ | –– | JQ975693 | –– |
|  | LSGB23404C8 | Ganyu, Jiangsu, China | GJ | –– | JQ975694 | –– |
|  | LSGB23404C9 | Ganyu, Jiangsu, China | GJ | –– | JQ975695 | –– |
|  | LSGB23404C3 | Ganyu, Jiangsu, China | GJ | –– | –– | JQ975788 |
|  | **?** | **Shantou, Guangdong, China** | **SG** | **GU393383** | **GU393372** | –– |
|  | **?** | **Shantou, Guangdong, China** | **SG** | **GU393384** | **GU393373** | –– |
|  | **?** | **Shantou, Guangdong, China** | **SG** | **GU393385** | **GU393374** | –– |
| ***N. acuminatus*** |  |  |  |  |  |  |
|  | **?** | **Zhauhai, Guangdong, China** | **ZHG** | **GU393380** | **GU393377** | –– |
|  | **?** | **Zhauhai, Guangdong, China** | **ZHG** | **GU393381** | –– | –– |
|  | **?** | **Zhauhai, Guangdong, China** | **ZHG** | **GU393382** | –– | –– |
| ***N.* sp1** | **?** | **Guanghai Bay, China** | **GC** | **GU393386** | **GU393375** | –– |
| ***N. algidus*** | **?** | **19°42’N, 112°29’ E** |  | **GU393388** | **GU393376** | –– |
|  | **?** | **19°42’N, 112°29’ E** |  | **GU393389** | –– | –– |
| ***N. castus*** | **?** | **?** |  | –– | **EU076710** | –– |
| ***N. semiplicatoides*** | **?** | **?** |  | –– | **EU076707** | –– |
| ***N. sufflatus*** | **?** | **Yangjiang Coast, China** | **YC** | **GU393387** | **GU393378** | –– |
| ***N. burchardi*** | **?** | **?** |  | **AY296837** | –– | –– |
| ***N. kraussianus*** | **?** | **?** |  | **DQ456981** | –– | –– |
| ***N. nitidus*** | **?** | **?** |  | **EF571481** | –– | –– |
| ***N. reticulatus*** | **?** | **?** |  | **EF571446** | –– | –– |
| ***N.* sp2** | **?** | **20**°**52’N, 114**°**53’ E** |  | **GU393390** | –– | –– |
| ***Fusinus longicaudus*** | **LSGB2341301** | **24**°**25’N, 118**°**07’ E** |  | **HQ834100** | **HQ833955** | –– |
| ***Euplica scripta*** | **LSGB23101** | **21**°**28’N, 109**°**07’ E** |  | **HQ834054** | **HQ833924** | –– |
| ***Mitrella burchardi*** | **LSGB23408** | **29**°**58’N, 122**°**12’ E** |  | **HQ834098** | **HQ833970** | –– |
| ***Pseudamycla formosa*** | **LSGB23409** | **29**°**58’N, 122**°**12’ E** |  | **HQ834097** | **HQ833969** | –– |
