# Supplementary material for: Monophyly, Distance and Character–Based Multigene Barcoding Reveal Extraordinary Cryptic Diversity in Nassarius: A Complex and Dangerous Community
Source: PLoS One. 2012 Oct 11;7(10):e47276. doi: 10.1371/journal.pone.0047276 (PMC3469534; doi:10.1371/journal.pone.0047276)
Supplement: Table S2 — The mean interspecific divergences of COI sequences. (DOC) [file pone.0047276.s002.doc]

Table S2. The mean interspecific divergences of COI sequences (lower left: nucleotide divergences, upper right: standard error)
