# Supplementary material for: Monophyly, Distance and Character–Based Multigene Barcoding Reveal Extraordinary Cryptic Diversity in Nassarius: A Complex and Dangerous Community
Source: PLoS One. 2012 Oct 11;7(10):e47276. doi: 10.1371/journal.pone.0047276 (PMC3469534; doi:10.1371/journal.pone.0047276)

Table S4: Character-based 16S rDNA barcodes for 19 defined clades of *Nassarius* in Figure 4; Character states (nucleotides) at 30 selected positions of the 16S rDNA gene region (ranging from position 114 – 426); Taxa name according to Tables S1 and Figure 4; Numbers of individuals analysed per species were given in brackets; Species showing cryptic diversity were marked in red; Species that could be identified as synonyms were marked in blue.


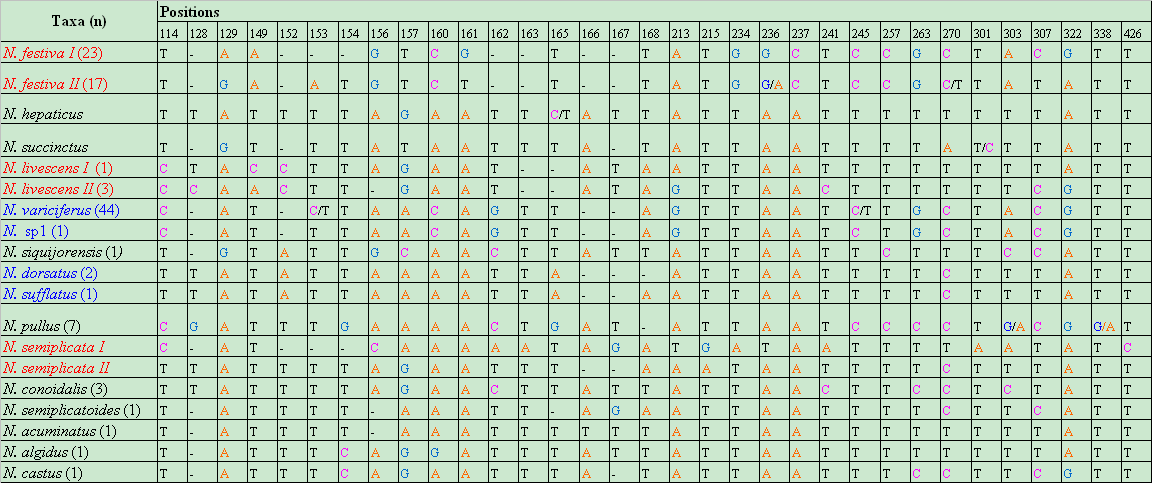

Supplement: Table S4 — Character-based DNA barcodes for 16S rDNA gene. (DOC) [file pone.0047276.s004.doc]
