# Supplementary material for: Monophyly, Distance and Character–Based Multigene Barcoding Reveal Extraordinary Cryptic Diversity in Nassarius: A Complex and Dangerous Community
Source: PLoS One. 2012 Oct 11;7(10):e47276. doi: 10.1371/journal.pone.0047276 (PMC3469534; doi:10.1371/journal.pone.0047276)

Table S5: Character-based ITS-1 barcodes for 11 defined clades of *Nassarius* in Figure 5; Character states (nucleotides) at 26 selected positions of the ITS-1 gene region (ranging from position 49 – 417); Taxa name according to Tables S1 and Figure 5; Numbers of individuals analysed per species were given in brackets.


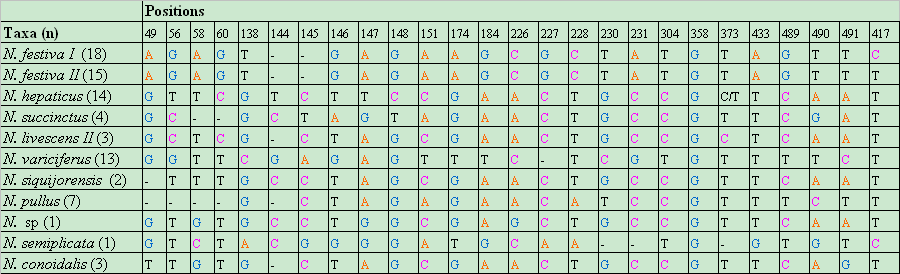

Supplement: Table S5 — Character-based DNA barcodes for ITS-1 gene. (DOC) [file pone.0047276.s005.doc]
